# Cu/Zn-superoxide dismutase and wild-type like fALS SOD1 mutants produce cytotoxic quantities of H_2_O_2_ via cysteine-dependent redox short-circuit

**DOI:** 10.1038/s41598-019-47326-x

**Published:** 2019-07-25

**Authors:** Shamchal Bakavayev, Nimrod Chetrit, Tatiana Zvagelsky, Rasha Mansour, Maria Vyazmensky, Zeev Barak, Adrian Israelson, Stanislav Engel

**Affiliations:** 1 Department of Clinical Biochemistry and Pharmacology, Faculty of Health Sciences, Ben-Gurion University of the Negev, Beer-Sheva, Israel; 2 Department of Chemistry, Faculty of Natural Sciences, Ben-Gurion University of the Negev, Beer-Sheva, Israel; 3 Department of Life Sciences, Faculty of Natural Sciences, Ben-Gurion University of the Negev, Beer-Sheva, Israel; 4 Department of Physiology and Cell Biology, Faculty of Health Sciences, Ben-Gurion University of the Negev, Beer-Sheva, Israel

**Keywords:** Enzyme mechanisms, Amyotrophic lateral sclerosis

## Abstract

The Cu/Zn−superoxide dismutase (SOD1) is a ubiquitous enzyme that catalyzes the dismutation of superoxide radicals to oxygen and hydrogen peroxide. In addition to this principal reaction, the enzyme is known to catalyze, with various efficiencies, several redox side-reactions using alternative substrates, including biological thiols, all involving the catalytic copper in the enzyme’s active-site, which is relatively surface exposed. The accessibility and reactivity of the catalytic copper is known to increase upon SOD1 misfolding, structural alterations caused by a mutation or environmental stresses. These competing side-reactions can lead to the formation of particularly toxic ROS, which have been proposed to contribute to oxidative damage in amyotrophic lateral sclerosis (ALS), a neurodegenerative disease that affects motor neurons. Here, we demonstrated that metal-saturated SOD1^WT^ (holo-SOD1^WT^) and a familial ALS (fALS) catalytically active SOD1 mutant, SOD1^G93A^, are capable, under defined metabolic circumstances, to generate cytotoxic quantities of H_2_O_2_ through cysteine (CSH)/glutathione (GSH) redox short-circuit. Such activity may drain GSH stores, therefore discharging cellular antioxidant potential. By analyzing the distribution of thiol compounds throughout the CNS, the location of potential hot-spots of ROS production can be deduced. These hot-spots may constitute the origin of oxidative damage to neurons in ALS.

## Introduction

The enzyme Cu/Zn-superoxide dismutase (SOD1) – a ubiquitous 32-kDa homodimeric protein – catalyzes the dismutation of superoxide radicals (O_2_∙^−^) to oxygen and hydrogen peroxide, and is critical for cell defense against reactive oxygen species (ROS)^1^. The Zn^2+^ ion plays a structural role and is required for an efficient SOD1 folding and long-term stability^2^, whereas active-site Cu^2+^ catalyzes O_2_∙^−^ dismutation by alternating between the reduced and oxidized states (Reactions 1 and 2):

1 $${\rm{S}}{\rm{O}}{\rm{D}}1-{{\rm{C}}{\rm{u}}}^{2+}+{{\rm{O}}}_{{2}^{\bullet }}^{-}\to {\rm{S}}{\rm{O}}{\rm{D}}1-{{\rm{C}}{\rm{u}}}^{+}+{{\rm{O}}}_{2}$$

2 $${\rm{S}}{\rm{O}}{\rm{D}}1-{{\rm{C}}{\rm{u}}}^{+}+{{\rm{O}}}_{{2}^{\bullet }}^{-}+2{{\rm{H}}}^{+}\to {\rm{S}}{\rm{O}}{\rm{D}}1-{{\rm{C}}{\rm{u}}}^{2+}+{{\rm{H}}}_{2}{{\rm{O}}}_{2}$$

In the nervous tissue, SOD1 is present at exceedingly high concentrations (~1% of total protein, i.e., 100–200 μM)^3^ and is traditionally regarded as cytosolic protein^4^. However, in cell culture experiments, the SOD1 secretory pathways were shown to account for substantial quantities of extracellular SOD1, reaching ~ 20% of the intracellular SOD1 level^5^.

Over 150 mutations in the SOD1 gene have been described to cause amyotrophic lateral sclerosis (ALS) – a fatal neurodegenerative disease that affects the upper and lower motor neurons^6,7^ – in an autosomal dominant fashion, accounting for about 20% of the familial cases of ALS (fALS)^8,9^. The phenotypic hallmark of the SOD1-dependent ALS is the presence of amyloid aggregates of SOD1 in affected tissues, which are the ultimate outcome of SOD1 misfolding – the product of a gradual destabilization of the SOD1 structure due to the loss of metal ions and the reduction of the stabilizing intrasubunit disulfide bond^10–12^. Although fALS mutations are known to accelerate SOD1 misfolding, their presence is not obligatory; evidence exists that environmental stresses, such as oxidative stress, can trigger noxious misfolding in the wild-type SOD1 (SOD1^WT^)^13–17^. SOD1^WT^ misfolding has been shown to occur in the spinal cord of sporadic (sALS) and non-SOD1 fALS patients^13,14,18–23^, and it was proposed that misfolded SOD1^WT^ and mutant proteins (SOD1^MUT^) share a common pathogenic pathway in ALS^18,23,24^.

A remarkable feature of the misfolded SOD1 is its prion-like ability to self-propagate, such that a misfolded SOD1 (either SOD1^WT^ or SOD1^MUT^) induces, via a yet-unknown mechanism, the misfolding of other, intact SOD1 molecules^15,25^. In ALS, the misfolding signal is believed to be transmitted via SOD1 secretory pathways in a cell-to-cell manner, thereby causing the disease to spread from the focal point of initiation throughout the spinal axis^5,22,25–30^. However, to date, the structural identity and the mechanism of neurotoxicity of the noxious SOD1 species remain obscure.

The fALS SOD1 mutants are categorized into wild-type-like (WTL), such as A4V, L38V, G37R, G41S, G72S, D76Y, D90A and G93A, that bind metals tightly and retain catalytic activity^31^, and those with impaired metal binding and reduced catalytic activity, such as H46R, H48Q, G85R, D124V, D125H, G127X and S134N^32^. The propensity to aggregate is one of the proposed noxious gain-of-functions of misfolded SOD1, although the relationships between aggregation and toxicity remain unclear. For instance, it was shown that the overexpression of fALS SOD1^G93A^ mutant and CCS1 Cu-chaperone in mice exacerbated neuronal damage with no detectable SOD1 aggregation^33^. The heterogeneity of the structural/functional properties of fALS SOD1 mutants prompts the quest of a common denominator, a factor whose presence may play role in the disease etiology. Because of the dominant character of SOD1-dependent ALS, SOD1^WT^ is present in the majority of fALS cases. Misfolding induced in the SOD1^WT^ by interaction with a fALS SOD1^MUT^ may confer the former toxic properties. An impaired redox chemistry catalyzed by the enzyme could be among such properties. Indeed, it has previously been demonstrated that in the SOD1^WT^ the loss of structural Zn^2+^, a common attribute of SOD1 misfolding^34^, increases accessibility of the active site to substrates other than superoxide and enhances oxidizing ability of the catalytic copper, thus facilitating ROS production^35,36^. Oxidative stress is a common characteristic of ALS pathogenesis^37–40^.

In spite of being generally regarded as antioxidants, many biological thiol compounds, including cysteine (CSH) and homocysteine (Hcy), are toxic to various cell types, in particular to neurons, both *in vitro* and *in vivo*^41–44^. The cytotoxic effect of thiols is often biphasic, as they are not toxic at either low (<0.1 mM) or high (>1 mM) concentrations^45^. The mechanism underlying thiol cytotoxicity is not entirely understood, but their oxidation in the presence of free Cu^2+^ ions to produce toxic ROS was proposed as a plausible possibility^45–50^. In this scenario, thiol compounds act as pro-oxidants. The active site of SOD1 is relatively solvent-exposed^2^ and the accessibility of the catalytic Cu^2+^ increases upon SOD1 misfolding^35,51,52^. Consequently, the SOD1 active-site copper has been demonstrated to catalyze oxidation of thiol compounds^53^.

Here we show that a metal-saturated SOD1^WT^ (holo-SOD1^WT^) and a fALS WTL mutant SOD1^G93A^ catalyze thiol oxidation, with CSH as a preferable substrate, to produce cytotoxic quantities of H_2_O_2_. GSH, the major cellular antioxidant contributing to both non-enzymatic and enzyme-dependent defense against ROS^37,38^, is an inefficient substrate for the SOD1-catalyzed H_2_O_2_ formation. However, in the presence of small quantities of CSH (or cystine), GSH becomes potent pro-oxidant that exacerbates the effect of CSH by donating reducing equivalents to regenerate free CSH. The resulting redox short-circuit, which is capable of draining GSH stores, requires sub-physiological GSH concentrations to operate, and may potentially be triggered by a combination of chronic conditions, such as aging, and an acute oxidative stress. Analyzing the distribution of the thiol compounds across the CNS indicated that, in astrocytes, the unique combination of GSH and CSH levels renders these cells a potential hot spot of ROS formation, from which, under certain pathophysiologic circumstances, neuronal damage may arise.

## Results

### Holo-SOD1^WT^ and WTL SOD1^G93A^ are cytotoxic in the presence of thiol compounds

**Figure 1:**
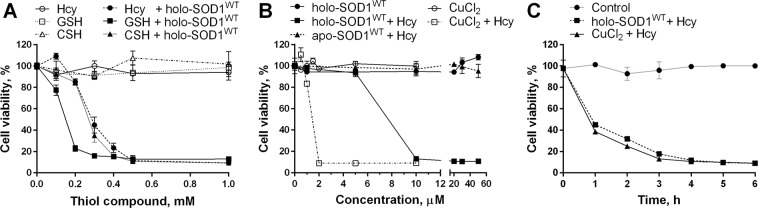
Thiol compounds induce holo-SOD1^WT^ cytotoxicity. (**A**) The viability of SH-SY5Y cells was determined after 6 h of incubation in the absence or presence of 50 μM (monomer-based) holo-SOD1^WT^ and the indicated concentrations of thiol compounds. (**B**) The viability of SH-SY5Y cells was determined after 6 h of incubation in the absence or presence of 0.5 mM Hcy and the indicated concentrations of holo-SOD1^WT^, apo-SOD1^WT^ or CuCl_2_. (**C**) Time course of Hcy-induced cytotoxicity. The viability of SH-SY5Y cells was determined at the indicated time points in the absence or presence of 0.5 mM Hcy and either holo-SOD1^WT^ (50 μM) or CuCl_2_ (5 μM). The viability of untreated cells was set to 100% (control). Results represent normalized means ± SD and are representative of at least three independent experiments performed in triplicates.

We tested the effect of CSH and other thiol compounds (namely, Hcy and GSH) on the viability of human neuroblastoma SH-SY5Y cells in the presence of extracellular holo-SOD1^WT^. When applied separately, neither holo-SOD1^WT^ nor thiol compounds were cytotoxic at any of the tested concentrations (Fig. 1A,B). Conversely, their simultaneous application resulted in cytotoxicity, whose extent depended on the concentration of both holo-SOD1^WT^ and the thiol compounds. Thiol cytotoxicity was not observed in the presence of metal-depleted SOD1^WT^ (apo-SOD1^WT^) (Fig. 1B).

**Figure 2:**
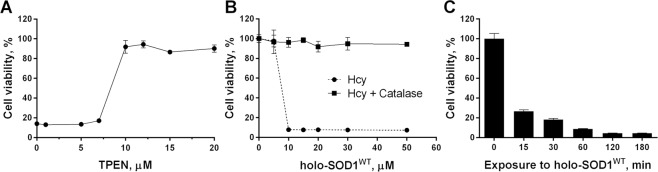
Thiol-dependent holo-SOD1^WT^ cytotoxicity is irreversible and can be prevented by Cu^2+^ chelator or by catalase. (**A**) The viability of SH-SY5Y cells was determined after 6 h of incubation in the presence of 10 μM (monomer-based) holo-SOD1^WT^, 0.5 mM Hcy, and the indicated concentrations of TPEN. (**B**) The viability of SH-SY5Y cells was determined after 6 h of incubation with or without 500 U/ml catalase in the presence of 0.5 mM Hcy and the indicated concentrations of holo-SOD1^WT^. (**C**) SH-SY5Y cells were incubated with 50 μM holo-SOD1^WT^ in the presence of 0.5 mM Hcy for the indicated periods of time, followed by further incubation in a fresh medium (without SOD1 or Hcy), and the viability was determined after 12 h of incubation in total. The viability of untreated cells was set to 100%. Results represent normalized means ± SD and are representative of at least three independent experiments performed in triplicates.

The fast kinetics of holo-SOD1^WT^-induced cell death in the presence of thiol compounds, as demonstrated by Hcy (Fig. 1C), suggest that the origin of cytotoxicity is extracellular, rather than due to the internalization of holo-SOD1^WT^ by the cells^54,55^. Moreover, separating holo-SOD1^WT^ from the cells using a 3-kDa cutoff membrane did not abolish cytotoxicity, although the kinetics of cell death were significantly delayed (Supplementary Fig. S1), demonstrating that cytotoxicity was facilitated by a diffusible low-molecular weight substance. The divalent metal ion chelator N,N,N’,N’-Tetrakis(2-pyridylmethyl)ethylenediamine (TPEN) abolished Hcy-induced holo-SOD1^WT^ cytotoxicity in a dose-dependent manner (Fig. 2A), implying that metal ions are involved in the mechanism responsible for this cytotoxicity.

**Figure 3:**
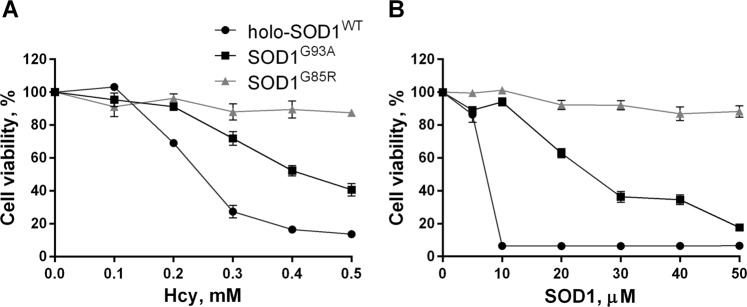
Wild-type-like SOD1^G93A^ but not metal-deficient SOD1^G85R^ mutant is cytotoxic in the presence of thiol compounds. (**A**) The viability of SH-SY5Y cells was determined after 6 h of incubation in the absence (control) or presence of 50 μM (monomer-based) metal reconstituted holo-SOD1^WT^, SOD1^G93A^ or SOD1^G85R^ and the indicated concentrations of Hcy. (**B**) The viability of SH-SY5Y cells was determined after 6 h of incubation in the absence (control) or presence of 0.5 mM Hcy and the indicated concentrations of holo-SOD1^WT^, SOD1^G93A^ or SOD1^G85R^. The viability of control cells was set to 100%. Results represent normalized means ± SD and are representative of at least three independent experiments performed in triplicates.

We also tested thiol-induced cytotoxicity in the presence of two fALS SOD1 mutants: WTL SOD1^G93A^ and metal binding-impaired SOD1^G85R^, which were purified and reconstituted with metals using the same procedure as the holo-SOD1^WT^ (see Methods). The SOD1^G93A^ demonstrated a reduced cytotoxic potency as compared to the holo-SOD1^WT^, while metal-deficient SOD1^G85R^ was essentially non-toxic at any of the tested concentrations of either Hcy or the protein (Fig. 3A,B).

### H_2_O_2_ is produced during the SOD1-catalyzed oxidation of thiol compounds

The toxicity of CSH and Hcy toward various cell types in the presence of free Cu^2+^ (as CuCl_2_) has been previously demonstrated and ascribed to the toxicity of H_2_O_2_ and/or of hydroxyl radicals produced during Cu^2+^-catalyzed thiols oxidation^46–50^. Although free Cu^2+^ was 5-fold more potent than holo-SOD1^WT^ in inducing cell death (Fig. 1B), the kinetics of the Hcy-dependent cell death were almost identical in both cases (Fig. 1C). Moreover, the cytotoxicity of Hcy was completely abolished by adding catalase at all examined concentrations of holo-SOD1^WT^ (Fig. 2B), indicating that this cytotoxicity was mediated by H_2_O_2_, and Cu^2+^ ions are involved in the mechanism of its formation. The luck of cytotoxicity in apo-SOD1^WT^ and metal-deficient SOD1^G85R^ is in line with this conclusion.

Extracellular H_2_O_2_ is an effective and potent neurotoxin, which can induce either apoptosis or necrosis in neurons^56–58^. Such cell death is associated with a burst-like increase in intracellular Ca^2+^ after a brief (several minutes) exposure to H_2_O_2_, as a result of the influx of Ca^2+^ from the extracellular space via redox-sensitive Ca^2+^ channels, such as TRPM2, which are expressed in neurons^59^. In agreement with this mechanism of neuronal death, a brief (<15 min) exposure of SH-SY5Y cells to extracellular holo-SOD1^WT^ in the presence of Hcy irreversibly damaged the cells (Fig. 2C).

**Figure 4:**
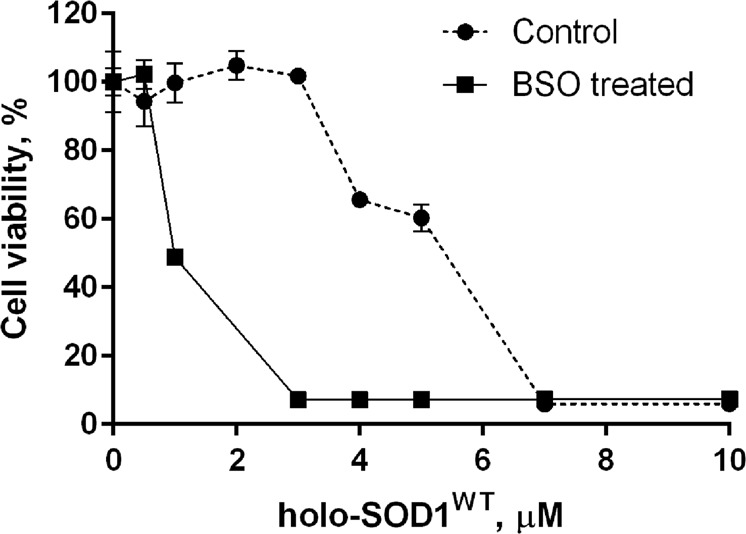
Cells pretreated with BSO are sensitized to holo-SOD1^WT^ cytotoxicity. The SH-SY5Y cells were pretreated for 18 h with or without (control) 1 mM BSO, followed by the addition of 0.3 mM Hcy and the indicated concentrations of holo-SOD1^WT^ (monomer based). The cells were incubated for additional 6 h and viability was determined as described in the Methods. Results represent normalized means ± SD and are representative of at least three independent experiments performed in triplicates.

Since the plasma membrane is permeable to H_2_O_2_^60^, extracellularly produced H_2_O_2_ may also aggravate existing oxidative stress conditions. Cells with an impaired ability to scavenge ROS are expected to be less resistant to an oxidative insult, including one of extracellular origin. Consistent with this idea, the cytotoxicity of holo-SOD1^WT^ in the presence of Hcy was significantly potentiated in SH-SY5Y cells pre-treated with buthionine sulfoximine (BSO), an inhibitor of glutathione synthesis^61^ (Fig. 4).

**Figure 5:**
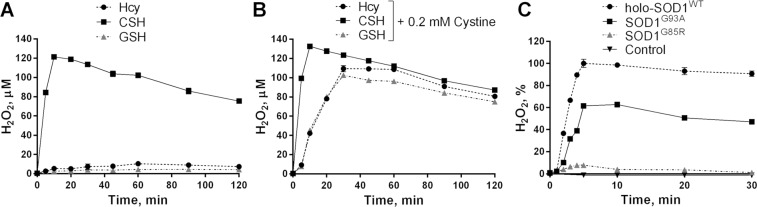
Metallated SOD1 catalyzes oxidation of thiol compounds to produce hydrogen peroxide. (**A**) Holo-SOD1^WT^ (20 μM) was incubated in 10 mM glycyl-glycine buffer, pH 7.5, 50 mM NaCl at 37 °C in the presence of 0.3 mM thiol compounds, and H_2_O_2_ concentration was measured at the indicated time points. (**B**) As in (A), but with the addition of 200 μM cystine. (**C**) Metal reconstituted holo-SOD1^WT^, SOD1^G93A^ or SOD1^G85R^ (20 μM) were incubated in the presence of 0.3 mM CSH, and H_2_O_2_ concentration was measured at the indicated time points. The maximal concentration of H_2_O_2_ produced by holo-SOD1^WT^ was set to 100%. Results represent means ± SD and are representative of at least three independent experiments performed in triplicates.

We then directly quantified the formation of H_2_O_2_ in the mixtures of SOD1 and thiol compounds using a phenol red/HRP H_2_O_2_ assay^62^. In cell experiments, all tested thiol compounds were toxic in the presence of holo-SOD1^WT^, with GSH being the most potent (Fig. 1A); in contrast, in a reconstituted reaction mixture, different rates of H_2_O_2_ formation were observed for different thiols (Fig. 5A and Supplementary Fig. S2). The fastest rates of oxidation were obtained with CSH, whereas Hcy was inferior and GSH failed to produce any significant amount of H_2_O_2_. Similar (but not identical) patterns of H_2_O_2_ production were observed in the presence of free Cu^2+^ (CuCl_2_) as a catalyst (Supplementary Fig. S2). In particular, the oxidation of Hcy in the presence of free Cu^2+^ exhibited a lag phase followed by a sigmoidal increase in the rate of H_2_O_2_ formation, whereas a monotonic asymptotic increase without lag phase was obtained in the presence of holo-SOD1^WT^ (Supplementary Fig. S2C,D).

The apparent discrepancy between the cytotoxic potencies of the thiol compounds and their respective rates of H_2_O_2_ formation in the reconstituted reaction mixture (in the presence of catalytically active SOD1) was reconciled when thiol oxidation was performed in the presence of cystine (oxidized cysteine dimer, CS-SC, 200 μM) – a component of the standard DMEM cell growth medium used in our cytotoxicity experiments. In the presence of cystine, all thiol compounds, including GSH, demonstrated similar rates of holo-SOD1^WT^-catalyzed H_2_O_2_ production (Fig. 5B), which were consistent with their cytotoxic potencies (Fig. 1A). Comparable rates of H_2_O_2_ formation were also observed when the measurements were performed directly in the DMEM cell growth medium (Supplementary Fig. S3).

**Figure 6:**
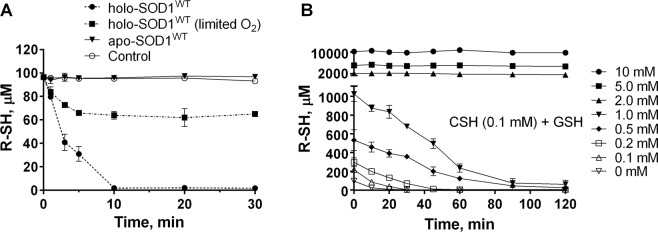
Cysteine-dependent redox short-circuit depletes GSH in the presence of holo-SOD1^WT^. (**A**) CSH (100 μM) was incubated at 37 °C in 20 mM Tris-HCl buffer, pH 7.5, 100 mM NaCl, in the absence (control) or presence of 10 μM holo-SOD1^WT^ or apo-SOD1^WT^. In a separate experiment the reaction mixture was purged with N_2_ for 1 h before holo-SOD1^WT^ addition. (**B**) The mixtures of CSH (100 μM) and the indicated concentrations of GSH were incubated in the presence of 10 μM holo-SOD1^WT^. The free thiol group concentration was measured at the indicated time points using DTNB reagent as described in the Methods. Results represent means ± SD and are representative of at least three independent experiments performed in triplicates.

We also monitored the kinetics of CSH substrate consumption in the course of H_2_O_2_ formation by using Ellman’s reagent (DTNB). The oxidation of CSH went to a completion in the presence of the holo-SOD1^WT^, while no significant thiol oxidation was observed with the metal-depleted apo-SOD1^WT^, Fig. 6A. As expected, the rate of CSH oxidation decreased under the conditions of limiting oxygen, and the reaction came to a halt when the remaining O_2_ was consumed.

In agreement with their respective cytotoxic potencies (Fig. 3), the WTL SOD1^G93A^ was by 40% less efficient in catalyzing H_2_O_2_ formation in the presence of CSH as compared to the holo-SOD1^WT^, whereas metal-deficient SOD1^G85R^ failed to produce any significant amounts of H_2_O_2_ (Fig. 5C). We suggest that the efficiency of thiol oxidation catalyzed by SOD1 is a function of its Cu^2+^ content, which in turn reflected the efficiency, with which Cu^2+^ was incorporation into the tested SOD1 variants upon the reconstitution (see Methods).

**Figure 7:**
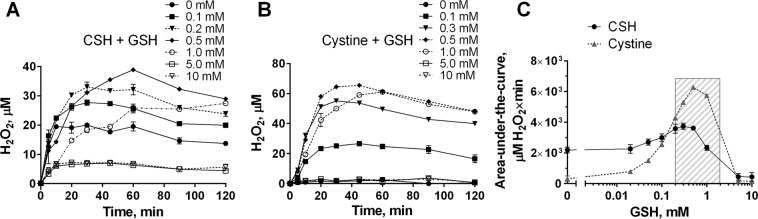
Glutathione promotes holo-SOD1^WT^-catalyzed H_2_O_2_ production via a cysteine-dependent redox short-circuit. Holo-SOD1^WT^ (20 μM) was incubated at 37 °C in 10 mM glycyl-glycine buffer, pH 7.5, 50 mM NaCl in the presence of 50 μM CSH (**A**) or cystine (**B**) and the indicated concentrations of GSH. The H_2_O_2_ concentration was measured at the indicated time points. In the absence of GSH, cystine does not produce H_2_O_2_. Results represent means ± SD and are representative of at least three independent experiments performed in triplicates. (**C**) The area under the curve of the time courses of H_2_O_2_ formation were calculated from (**A**) and (**B**) (between 0 and 120 min) using Prism 6 software (GraphPad) and plotted against GSH concentration. The shaded area represents the region, in which oxidative stress may occur. Results represent means ± SE calculated from two independent experiments performed in triplicates.

Although GSH cannot be oxidized directly by SOD1 to produce H_2_O_2_, it is apparently capable of reducing cystine to regenerate free CSH: 2GSH + CS-SC → GS-SG + 2CSH^53,63^. The CSH that is thereby produced enters another cycle of oxidation. Similarly to GSH, Hcy is capable of regenerating CSH from cystine^53,63^ (Fig. 5A,B). The total amount of H_2_O_2_ produced by a given amount of CSH or cystine was proportional to the amount of GSH present in the system (Fig. 7). The dependence of H_2_O_2_ accumulation on GSH concentrations, however, was bell-shaped, demonstrating that H_2_O_2_ buildup was inhibited at high GSH concentrations (Fig. 7C).

To distinguish between the possibilities that the inhibitory effect of high GSH concentrations was caused by the ability of the thiol compounds (including GSH) to effectively scavenge H_2_O_2_ in a non-enzymatic Cu^2+^-independent process (2GSH + H_2_O_2_ → GS-SG + 2H_2_O)^64^, or alternatively, by the inhibition of H_2_O_2_ *formation*, we monitored the kinetics of free thiol oxidation in the CSH/GSH mixtures in the presence of holo-SOD1^WT^, Fig. 6B. Although GSH alone was resistant − at any of the tested concentrations − to the holo-SOD1^WT^-catalyzed oxidation (not shown), in the presence of small quantities of CSH (100 μM), GSH was rapidly and completely oxidized, demonstrating a marked ability of the redox short-circuit thus established to deplete GSH stores. At the GSH concentrations higher than 2 mM, however, no significant GSH oxidation was observed, consistent with the lack of H_2_O_2_ formation at high GSH concentrations (Fig. 7). The mechanism responsible for the inhibitory effect of high GSH concentrations on the rate of H_2_O_2_ formation is unclear, but it may involve a competitive inhibition of CSH binding to the SOD1 active-site by GSH^65^; this competition, however does not prevent much smaller substrates, such as superoxide, from accessing the catalytic site, therefore enabling the enzyme’s action at the physiological GSH concentrations.

### Thiol compounds increase the accessibility of Cu^2+^ to external chelators

**Figure 8:**
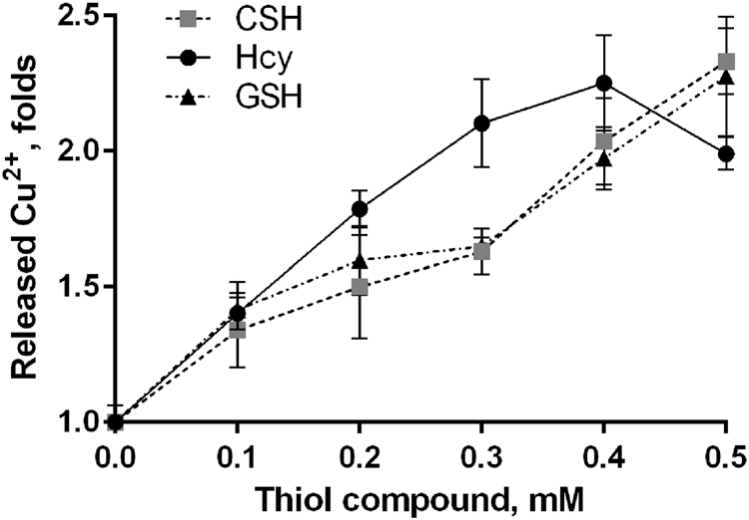
Thiol compounds increase the accessibility of SOD1 copper to exogenous chelator. Holo-SOD1^WT^ (50 μM) was incubated for 20 h at 37 °C with 100 μM PAR in the presence of the indicated concentrations of thiol compounds. PAR absorbance was measured at 490 nm and 520 nm, and the concentrations of released (PAR-complexed) metals were calculated as described in the Methods. The results were normalized to the amount of metals released in the absence of thiol compounds (fold change, mean ± SD) and are representative of at least three independent experiments performed in triplicates.

As high-affinity binding of metals to SOD1 is characterized by fast association–dissociation dynamics, strong chelators may interfere with the equilibrium^2^. To test whether thiol compounds increase the accessibility of holo-SOD1^WT^ metals to external chelators, we used as chelator the colorimetric divalent metal sensor 4-(2-pyridylazo)resorcinol (PAR)^36^. The incubation of holo-SOD1^WT^ with PAR in the presence of thiol compounds resulted in an increased formation of PAR-copper complexes (Fig. 8), while virtually no effect of thiol compounds was observed on the rate of PAR-Zn^2+^ complex formation (not shown).

**Figure 9:**
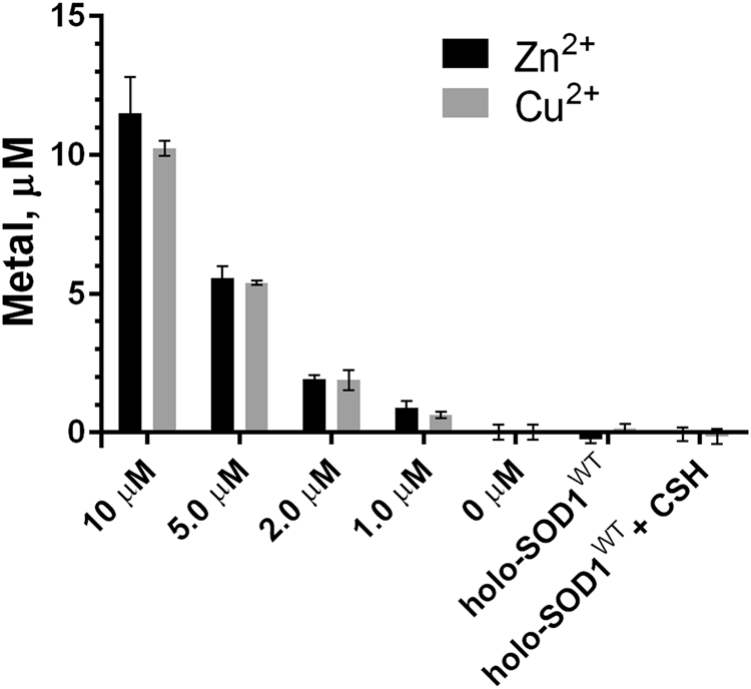
Thiol compounds form no binary complexes with SOD1^WT^-derived copper. Holo-SOD1^WT^ (100 μM) was incubated in the absence or presence of 0.5 mM CSH at 37 °C in 10 mM glycyl-glycine buffer, pH 7.5, 50 mM NaCl for 15 min (corresponding to the peak of H_2_O_2_ formation, Fig. 3). The reaction mixture was separated from the protein by ultrafiltration, and the filtrate was analyzed for the presence of labile metal ions after the complete oxidation of the remaining thiols (10 h at 37 °C) by adding 100 μM PAR. Metal standards (0–10 μM) were added to separate reaction mixtures to calibrate the system. The concentration of metals was determined as described in the Methods.

The mechanism by which the thiol compounds increase the accessibility of PAR to SOD1 copper is unknown. It is possible that thiol compounds reduce Cu^2+^ to Cu^+^ directly in the active site of SOD1^53^, thus changing its coordination geometry to non-tetrahedral and facilitating ligand replacement (PAR binding)^52^. In addition, the formation of a binary thiol – Cu^+^ complex could increase copper accessibility to PAR. The latter possibility, however, appears unlikely, since in line with the assumption that the thiol oxidation is catalyzed by the enzyme-bound Cu^2+^, CSH (similarly to other thiols) was incapable of extracting metal ions (Cu^2+^ or Zn^2+^) from the active site of holo-SOD1^WT^ (Fig. 9). The filtrate of the reaction mixture containing 100 μM holo-SOD1^WT^ and 0.5 mM CSH was analyzed for the metal presence using PAR. No labile metal ions were detected in the filtrate after the complete oxidation of the remaining thiols, indicating that, during the catalysis, no soluble binary complexes between the thiol compound and SOD1-derived Cu^2+^ were formed.

### Thiol oxidation promotes the formation of the intramolecular disulfide in holo-SOD1^WT^

**Figure 10:**
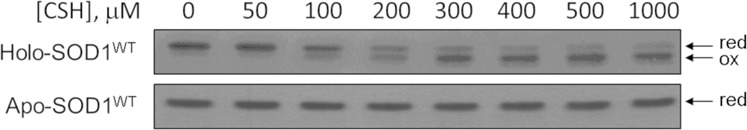
Cysteine promotes disulfide bond formation in metallated SOD1^WT^. Holo-SOD1^WT^ or apo- SOD1^WT^ (50 μM) were fully reduced by DTT (5 mM) and then exposed to the indicated concentrations of CSH for 30 min at 37 °C. After blocking free cysteine groups with iodoacetamide, the protein was separated by a non-reducing 12% SDS-PAGE. Data are representative of three independent experiments. Full-length gels are presented in Supplementary Figure S4.

The H_2_O_2_ produced during holo-SOD1^WT^-catalyzed thiol oxidation would increase the oxidizing potential of the protein’s immediate environment, potentially affecting redox-sensitive surface-exposed groups of the protein. The SOD1 contains a highly conserved intramolecular disulfide bond (Cys^57^-Cys^146^), which is required for the long-term stability and full catalytic activity of SOD1^66^. The formation of this bond in newly synthesized SOD1 is facilitated by CCS1 Cu-chaperone and is coupled to copper insertion^66,67^. The process is initiated by a copper-mediated (and CCS1-independent) oxidative step, in which one of the SOD1 cysteine residues becomes sulfenylated. The sulfenylation is subsequently resolved, in the presence of CCS1, to form a stable disulfide bond^66^. This disulfide, which is surface-exposed, is rather unusual feature for the protein found predominantly in the highly reducing cytosolic environment^66–68^. We tested the effect of holo-SOD1^WT^-catalyzed CSH oxidation on the redox status of the SOD1 intramolecular disulfide bond (Fig. 10). The holo-SOD1^WT^ or apo-SOD1^WT^ were initially reduced by a high concentration of DTT (5 mM) and then exposed to the increasing concentrations of CSH (0–1 mM). After blocking free cysteine groups with iodoacetamide to prevent disulfide bond scrambling, the disulfide status of the proteins was analyzed using a non-reducing SDS-PAGE^69,70^. Counterintuitively, considering the cysteine’s status as cellular reductant, increasing concentrations of CSH resulted in a progressively *increasing* proportion of holo-SOD1^WT^, but not apo-SOD1^WT^, containing oxidized disulfide bond (Fig. 10). We therefore concluded that the disulfide bond formation in the metallated SOD1 was facilitated, in the *absence* of CCS1, by ROS generated via the active-site Cu^2+^-catalyzed CSH oxidation. Understanding the physiological significance of such ability of metallated SOD1^WT^ to ‘self-repair’ its disulfide bond in the presence of ubiquitous biological thiols, and how this ability is affected by fALS mutations, requires further investigation. It is tempting to speculate, however, that the ROS-producing reaction may contribute to the long-term stability of SOD1 by preserving the oxidized status of its conserved disulfide in a highly reducing atmosphere of cellular interior.

## Discussion

The active-site Cu^2+^ of holo-SOD1^WT^ and fALS WTL SOD1^G93A^ mutant is capable of catalyzing the oxidation of various thiol compounds, including CSH and Hcy, with a concomitant production of H_2_O_2_. Conversely, the ubiquitous cellular thiol antioxidant GSH is resistant to the SOD1-catalyzed oxidation. In the presence of small quantities of CSH or cystine, however, GSH becomes potent pro-oxidant that fuels the CSH-dependent H_2_O_2_ formation by reducing cystine back to CSH. The GSH/CSH mixtures, therefore, may constitute a potent redox short-circuit that, under certain pathophysiological metabolic circumstances, could drain − in the presence of metallated SOD1 − GSH stores and, thereby, discharge the antioxidant potential of the cell.

Further investigation is required to elucidate the significance of the SOD1-catalyzed thiol oxidation with the concomitant H_2_O_2_ production to ALS pathogenesis. The reaction involves catalytic copper and as such it characterizes both holo-SOD1^WT^ and WTL fALS SOD1 mutants. Conversely, metal-deficient fALS mutants lack this ability, which raises the possibility that the described reaction is a peculiar catalytic ability of metallated SOD1 and it may not be related to ALS. Alternatively, one may hypothesize that ALS is facilitated by a synergistic interplay between a fALS SOD1 mutant and SOD1^WT^. According to this scenario, a catalytically inactive SOD1 mutant may act as a prion-like transmitter of the misfolding signal to efficiently spread the pathology, whereas the neurotoxic effect *per se* is caused by the ubiquitous SOD1^WT^, the structural and catalytic properties of which compromised by its interaction with the misfolded SOD1 mutant.

Under the assumption that the described mechanism of H_2_O_2_ production by thiol oxidation contributes to ALS pathogenesis, two important questions need to be addressed. The first one is whether the metabolic conditions required for the CSH-dependent short-circuit of GSH oxidation exist in CNS. A significant fraction of the cellular SOD1 may reach the extracellular space^5^. Although the cytotoxicity of extracellular SOD1 may represent a plausible mechanism of pathogenesis in disorders characterized by high concentrations of extracellular thiols (e.g., in homocystinuria, where plasma Hcy may reach 500 μM^71^), it is unlikely the pathogenic mechanism in ALS. This is because the extracellular, as demonstrated by CSF, concentrations of both GSH and CSH (or cystine) are very low^72–77^. Although the *intracellular* concentration of GSH in neurons is high^77–79^, the CSH content is very low (under the detection limit^77^) and, therefore, an efficient CSH-dependent GSH oxidation is unlikely in these cells. In astrocytes, by contrast, the intracellular content of GSH is one of the highest among mammalian cells (8–10 mM)^77–79^, and the level of CSH is substantial^80^. Supplemented with the ubiquitously expressed SOD1^81^, this combination renders astrocytes a possible locale for the CSH-dependent short-circuiting of GSH oxidation to produce high quantities of H_2_O_2_.

In healthy astrocytes (8–10 mM GSH) a bell-shaped dependency of H_2_O_2_ formation on GSH concentration (Fig. 7C) would effectively prevent H_2_O_2_ accumulation. However, under certain pathophysiologic circumstances, including aging, the total glutathione and/or GSH/GS-SG ratio may drop significantly^38–40^. According to the bell-shaped dependence curve, this change may result in the production of substantial quantities of H_2_O_2_ (shaded area in Fig. 7C). The increased formation of ROS may compromise the neuro-supportive (neuro-protective) potential of astrocytes resulting in neurotoxicity^82–91^.

The second question is as to how misfolding may affect the SOD1 catalytic properties to facilitate disease. H_2_O_2_ is considered a relatively benign ROS due to the presence of H_2_O_2_-detoxifying enzymes, such as catalase and GSH peroxidases (although the performance of the latter is compromised by low GSH concentrations). It was previously demonstrated that SOD1 can utilize H_2_O_2_ as a sole substrate to produce hydroxyl radical^51,92,93^, a highly potent ROS that targets lipids, sugars, DNA bases, amino acids, and organic acids^38,94^, and against which no enzymatic defense exists. In this process, a reduction of SOD1 Cu^2+^ by H_2_O_2_ (Reaction 3) is followed by a Cu^+^ oxidation by another H_2_O_2_ in a Fenton-type reaction to generate a hydroxyl radical (Reaction 4)^51,92,93^:

3 $${\rm{SOD}}1-{{\rm{Cu}}}^{2+}+{{\rm{H}}}_{2}{{\rm{O}}}_{2}\to {\rm{SOD}}1-{{\rm{Cu}}}^{+}+{{\rm{O}}}_{{2}^{\bullet }}^{-}+2{{\rm{H}}}^{+}$$

4 $${\rm{SOD}}1-{{\rm{Cu}}}^{+}+{{\rm{H}}}_{2}{{\rm{O}}}_{2}\to {\rm{SOD}}1-{{\rm{Cu}}}^{2+}+{{\rm{HO}}}^{\cdot }+{{\rm{OH}}}^{-}$$

The hydroxyl radical formation is markedly accelerated in the fALS SOD1 mutants, as compared with the native SOD1^WT^, a feature attributed to structural instability of the former manifested in the increased openness and accessibility of the active-site Cu^2+^ to substrates other than superoxide^35,51,95^. However, due to the low affinity of H_2_O_2_ to the SOD1 active site, non-physiologically high concentrations of H_2_O_2_ (10–20 mM) were required to produce substantial amounts of HO∙ ^92^, questioning the significance of this reaction to ALS pathogenesis. It was proposed that the HO∙ formation in the presence of H_2_O_2_ could, in principle, be facilitated by a cellular reductant other than H_2_O_2_ (whose identity remains unknown) capable of activating the active-site Cu^2+^ ^35,51^, therefore decreasing the amount of H_2_O_2_ required to produce HO∙ ^57,59^. We speculate that ubiquitous thiol compounds may play the role of such activating substance. Moreover, during thiol oxidation, H_2_O_2_ substrate of the second step (Reaction 4) is generated by SOD1 itself, thus the effective local concentration of H_2_O_2_ near the enzyme’s active-site is high. It could be especially true when considering the abnormal tendency of misfolded SOD1 to accumulate on particular locations in the cell^96,97^, hence further increasing local H_2_O_2_ concentration and accelerating the reaction by mass action.

Since the thiol oxidation by SOD1 is insensitive to the presence of Mn-SOD, it was concluded that superoxide is not formed as intermediate in this reaction^53^. Such resistance to the Mn-SOD presence, however, might results from the differences in the kinetics of the competing reactions or because peroxide may not be released as a free intermediate^35^. If superoxide is generated during the catalysis, it may react with NO to produce peroxynitrite, a powerful and much more (10,000 folds) stable oxidant than the hydroxyl radical^38,98–100^. Peroxynitrite exhibits a broad range of tissue-damaging effects, including lipid peroxidation, enzyme and ion channel inactivation, and inhibition of mitochondrial respiration,^98^ and the decomposition of peroxynitrite produces hydroxyl radicals^101^. It has been demonstrated that, in the absence of structural Zn^2+^, the coordination of Cu^2+^ in the active site of SOD1 changes, rendering Cu^2+^ a much more potent oxidant^35,36^. The Zn^2+^-deficient/Cu^2+^-SOD1^WT^ and fALS WTL SOD1 mutants were shown to efficiently oxidize ascorbate, with a subsequent reoxidation of SOD1-Cu^+^ by oxygen to produce O_2_∙^−^, which combines with NO afterward to produce peroxynitrite^35^. We speculate that the peroxynitrite production by misfolded metallated SOD1 may benefit, similarly to the HO∙ formation, by the ability of SOD1 enzyme to accept ubiquitous biological thiols as an alternative substrate for cooper activation.

## Methods

### Purification of recombinant SOD1^WT^, SOD1^G93A^ and SOD1^G85R^ proteins and their reconstitution with metals

The human SOD1^WT^, SOD1^G93A^ and SOD1^G85R^ were produced in *E. coli* BL-21 grown *without* Zn^2+^ and Cu^2+^ supplements and purified to homogeneity under non-denaturing conditions, as described previously^102^. The MALDI-TOF mass spectrometry analysis of SOD1^WT^ (Autoflex™ speed MALDI TOF/TOF mass spectrometer, Bruker, Germany) (Supplementary Fig. S5) and the purified protein’s low enzymatic activity 3.1 ± 0.2 U/mg, determined by a superoxide dismutase assay kit (Cayman Chemical, Ann Arbor, MI), indicated that this protein is demetallated (apo-SOD1^WT^) or metallated only partially. As shown previously, the recombinant SOD1^WT^ produced in *E. coli* and reconstituted with metals by a dialysis *after* purification, regains its full catalytic activity and could be regarded as holo-SOD1^WT^ ^103–105^. Indeed, we showed that saturation with Zn^2+^ and Cu^2+^ by the dialysis increased the catalytic activity of the recombinant SOD1^WT^ by three order of magnitude (1200 ± 40 U/mg) to approach that of the SOD1 standard provided with the SOD1 activity assay kit (3700 ± 200 U/mg). The MALDI-TOF analysis revealed that the average molecular mass difference between the metallated and apo-SOD1^WT^ is consistent, within the instrument’s sensitivity limit, with the addition of two metal ions per SOD1 monomer: 108 Da difference for the monomer peak (expected 129 Da) and 280 Da difference for the dimer peak (expected 258 Da), Supplementary Fig. S5.

Specifically, the purified SOD1 proteins were reconstituted with metals by an overnight dialysis against buffer A (50 mM Tris-HCl, pH 7.5, 0.1 M NaCl, and 10% glycerol) supplemented with ZnSO_4_ and CuSO_4_ (1 mM each). The dialyzed proteins were incubated for 1 h at 4 °C in the presence of 3 mM EDTA to chelate unbound and weakly bound metals, and then dialyzed overnight against buffer A. The dialyzed proteins were further buffer-exchanged using five 1:4 dilution (buffer A) and ultrafiltration (10 kDa cutoff, Amicon, Millipore, Burlington, MA) steps to remove residual metals and EDTA. The protein was then centrifuged at 110,000 × *g* for 1 h at 4 °C using an ultracentrifuge (Sorvall M120, Discovery, Thermo Fisher Scientific, UK) and stored, at the concentration 30 mg/ml, at −20 °C under argon until used. Protein concentration was measured by the Bradford method, using bovine serum albumin (fatty acid free, Sigma-Aldrich, Israel) as standard, and spectroscopically using a monomeric molar extinction coefficient at 280 nm of 5500 M^−1^·cm^−1^ (the two methods produced similar estimations).

### Cell viability assay

SH-SY5Y cells were seeded in 96-well plates at a starting density of 1.5 × 10^4^ cells/well in a high glucose Dulbecco’s modified Eagle medium (DMEM) supplemented with 1% L-glutamine, 1% penicillin-streptomycin, and 10% (vol/vol) fetal bovine serum (FBS), and incubated for 10 h prior experiments. The experiments were performed in the medium containing 2% FBS. At the end of the experiment, the cells were washed once with Hank’s balanced salt solution (HBSS) and cell viability was assessed by adding 20 µL of the CellTiter 96 AQueous One Solution reagent (Promega, Fitchburg, WI) to 100 μl HBSS, followed by incubation for 60 min at 37 °C, and the absorbance was measured at 490 nm using the Infinite 200 PRO plate-reader (Tekan, Switzerland). We found that mycoplasma infection decreased the cells susceptibility to SOD1 cytotoxicity; therefore, the cells were treated for two weeks prior the experiments with 25 μg/ml Plasmocin (Invitrogen, Sweden). We also found that the magnitude of the cytotoxic effect of SOD1 was inversely proportional to the cell density; therefore, the cell seeding conditions were adjusted to perform all the experiments at the final confluency of 70%.

In the compartmentalization experiments, SOD1 was separated from the cells cultured in a 24-well plate using a tightly fit dialysis insert equipped with a 3.5-kDa cutoff membrane (Thermo Fisher Scientific, UK).

### Colorimetric metal assay

The holo-SOD1^WT^ (50 μM, monomer based) was incubated for 20 h at 37 °C in 50 mM Hepes buffer, 50 mM NaCl, pH 7.4, in the presence of the indicated concentrations of thiol compounds and 100 μM divalent metal dye 4-(2-pyridylazo)resorcinol (PAR), in 90 μl total volume in 384-well plates covered with a transparent adhesive film (to prevent evaporation). The absorbance was measured at 490 nm and 520 nm using the Infinite 200 PRO plate-reader (Tekan, Switzerland) and the concentrations of dye-complexed copper and zinc ions were determined using the method described by Mulligan *et al*.^106^.

To determine the concentration of metals present in solution, 100 μM holo-SOD1^WT^ were incubated for 15 min at 37 °C in 10 mM glycyl-glycine buffer, pH 7.5, 50 mM NaCl, in the absence or presence of 0.5 mM CSH. At the end of incubation, the protein was separated from the reaction mixture by ultra-filtration (5 kDa cutoff, Vivaspin 500, Sartorius, Germany) and the filtrate was analyzed for the presence of metals after a complete oxidation of the remaining thiols (10 h at 37 °C) by adding 100 μM PAR, as described above.

### H_2_O_2_ assay

H_2_O_2_ was determined according to the method described by Pick *et al*.^107^ with some modifications. Briefly, a reagent solution containing 0.56 mM phenol red (Alfa Aesar, UK) and 17 U/ml horseradish peroxidase (Type II, Sigma-Aldrich, Israel) in 10 mM glycyl-glycine buffer, pH 7.5, 50 mM NaCl, was added at a 1:1 volumetric ratio to the analyzed sample, and the mixture was incubated at room temperature for 2 min (5 min produced similar results), followed by the addition of NaOH to the final concentration of 50 mM to reach pH 12.5. The absorbance was measured at 610 nm using the Infinite 200 PRO plate-reader (Tekan, Switzerland).

### Free thiol assay

The analyzed samples were incubated for 15 min at room temperature in 20 mM Tris-HCl buffer, pH 7.5, NaCl 100 mM, 1 mM EDTA, 5 % DMSO in the presence of  0.5 mM Ellman’s Reagent (DTNB, 5,5′-Dithio-bis-(2-nitrobenzoic acid), Sigma-Aldrich, Israel), and the absorbance was measured at 412 nm using the Infinite 200 PRO plate-reader (Tekan, Switzerland).

### Holo-SOD1^WT^ disulfide bond status assay

The holo-SOD1^WT^ or apo-SOD1^WT^ (50 μM) were incubated for 0.5 h at 37 °C in 20 mM Tris buffer, pH 7.5, 0.1 M NaCl in the presence of 5 mM DTT to reduce the protein’s disulfide bonds. The residual DDT was removed from the reaction mixture by a buffer exchange using an ultrafiltration unit (10 kDa cutoff, modified PES, VWR International, USA). The fully reduced SOD1 was then incubated for 30 min at 37 °C in 20 mM Tris buffer, pH 7.5, 0.1 M NaCl in the presence of the indicated concentrations of CSH. The reaction mixture containing 0.8 mg/ml protein was then mixed (1:3, volumetric) with a SDS gel-loading solution (8% SDS, 40% Glycerol, 160 mM Tris-HCl, pH 6.8) and heated for 20 min at 65 °C. At the end of incubation, the protein was reacted at r.t with 20 mM iodoacetamide for 1 h and then separated by a non-reducing 12% SDS-PAGE. The gel was stained with Coomassie Brilliant Blue (Supplementary Fig. S4).

All data analyses were performed using the GraphPad Prism 6.0 software.

## Supplementary information

Supplementary Figures S1-S5
