# Supplementary material for: Cu/Zn-superoxide dismutase and wild-type like fALS SOD1 mutants produce cytotoxic quantities of H2O2 via cysteine-dependent redox short-circuit
Source: Sci Rep. 2019 Jul 25;9:10826. doi: 10.1038/s41598-019-47326-x (PMC6658568; doi:10.1038/s41598-019-47326-x)
Supplement: Supplementary file 1 — Supplementary Figures S1-S5 [file 41598_2019_47326_MOESM1_ESM.pdf]

Cu/Zn-superoxide dismutase and wild-type like fALS SOD1 mutants produce cytotoxic quantities of H_2O_2 via cysteine-dependent redox short-circuit.

Shamchal Bakavayev, Nimrod Chetrit, Tatiana Zvagelsky, Rasha Mansour, Maria Vyazmensky, Zeev Barak, Adrian Israelson, and Stanislav Engel

Supplementary Figures

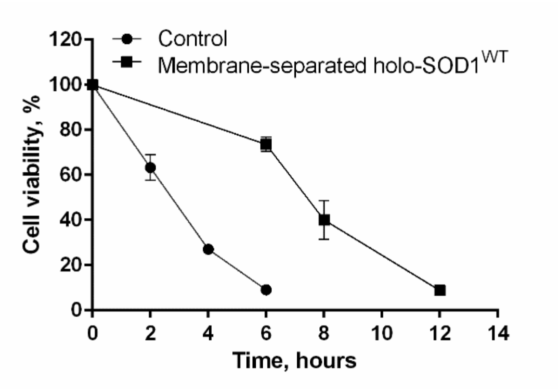

Supplementary Figure S1. Low-molecular weight substance(s) mediate the thiol-dependent cytotoxicity of holo-SOD1^{WT}. The SH-SY5Y cells grown in a 24-well plate were exposed to holo-SOD1^{WT} applied either directly (50 μM) or separated from the cells by a 3.5 kDa cutoff membrane (120 μM holo-SOD1^{WT} were placed in a micro-dialysis insert [ThermoFisher Scientific, UK] fit into the well). The indicated concentrations were used to keep the total amount of holo-SOD1^{WT} equal in both experiments. The viability of the cells was measured at the indicated time points. Results represent normalized means ± SD and are representative of at least three independent experiments performed in duplicates.

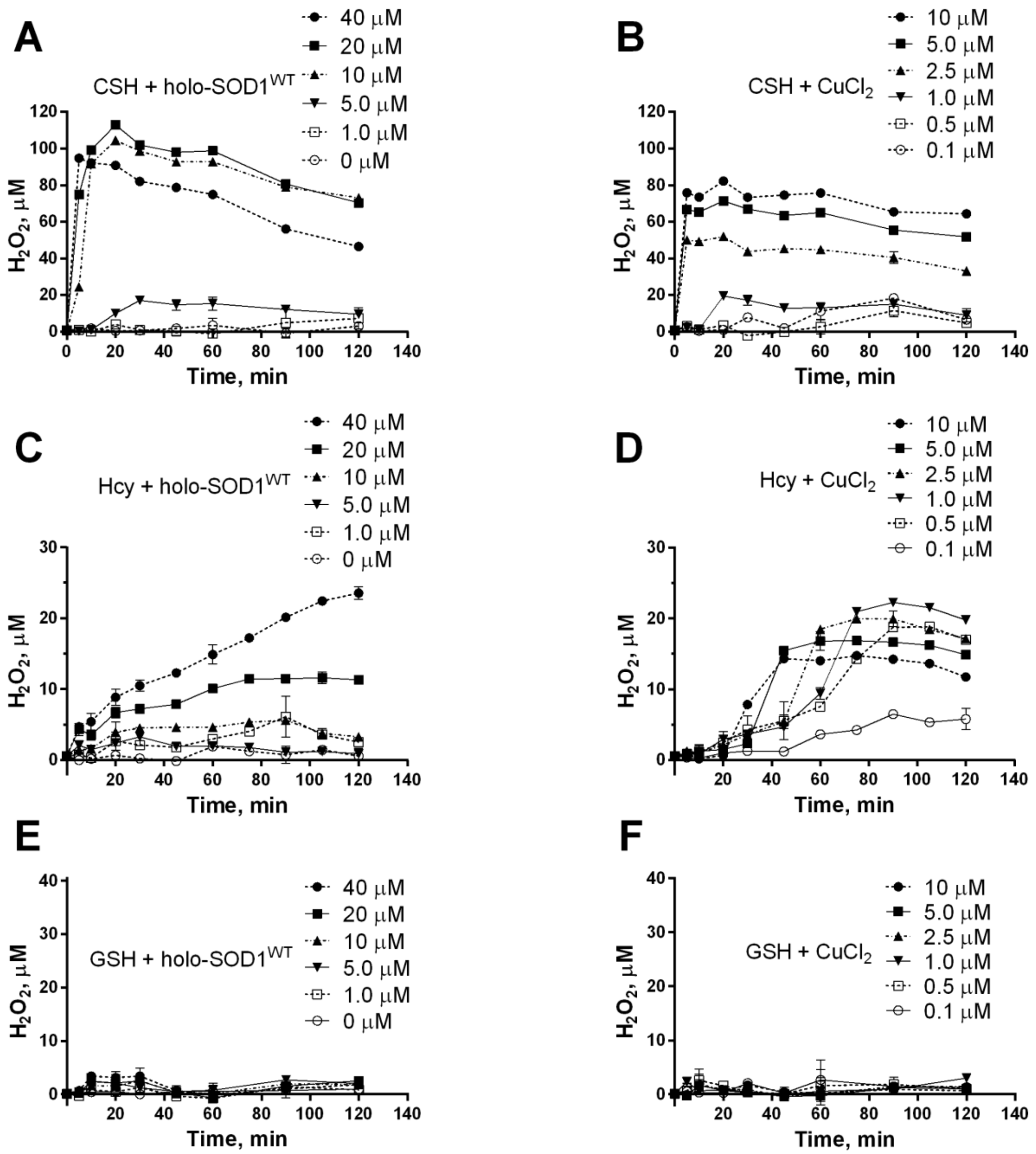

Supplementary Figure S2. Holo-SOD1^{WT} and free Cu^{2+} catalyze H_2O_2 production via oxidation of thiol compounds. The indicated concentrations of holo-SOD1^{WT} (**A, C, E**) or free Cu^{2+} (as $CuCl_2$) (**B, D, F**) were incubated at 37 °C in 10 mM glycyl-glycine buffer, pH 7.5, 50 mM NaCl in the presence of 300 μM CSH (**A, B**), Hcy (**C, D**) or GSH (**E, F**), and H_2O_2 concentration was measured at the indicated time points. Results represent means ± SD and are representative of at least three independent experiments performed in triplicates.

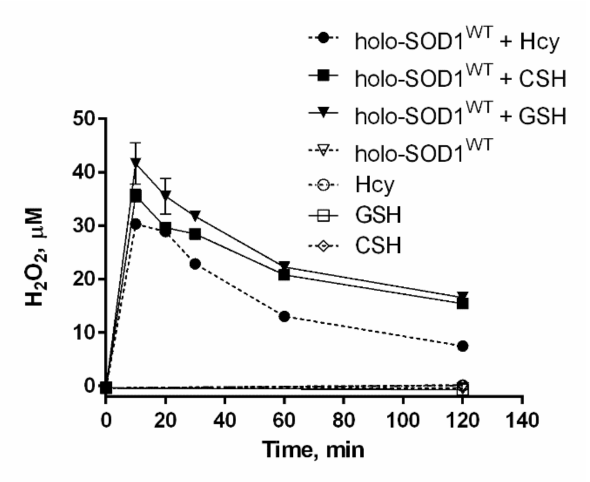

Supplementary Figure S3. CSH, Hcy and GSH are equally efficient in producing H_2O_2 in DMEM. 300 μM CSH, Hcy or GSH were incubated with or without holo-SOD1^{WT} at 37 °C in the DMEM growth medium (Biological Industries, Israel, contains 200 μM cystine) supplemented with 10 mM Na^+-phosphate buffer, pH 7.5, and 2% FBS, and H_2O_2 concentration was measured at the indicated time points. Results represent means ± SD and are representative of at least three independent experiments performed in triplicates.

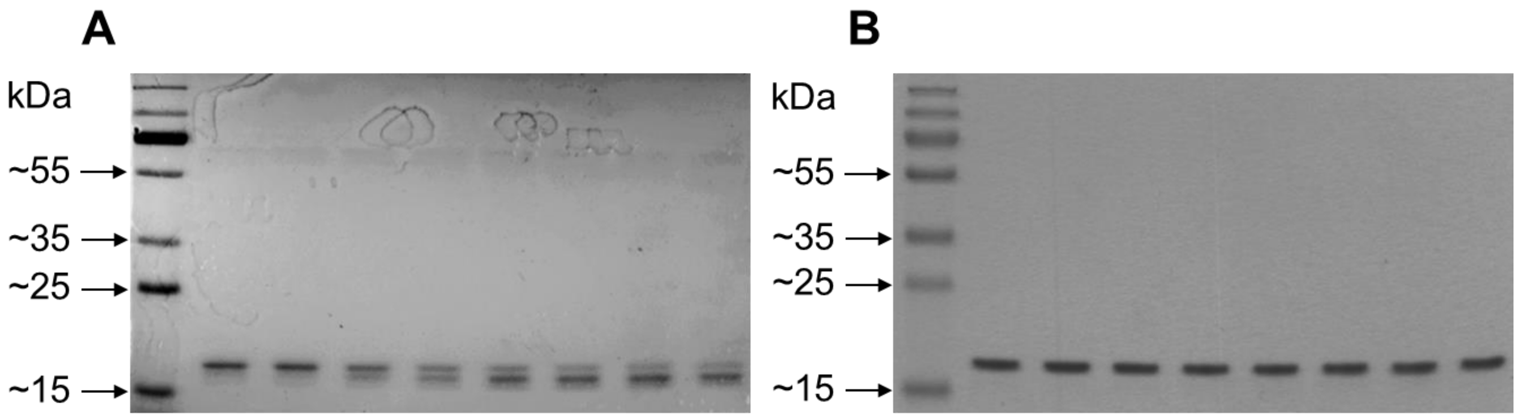

Supplementary Figure S4. Cysteine promotes disulfide bond formation in metallated SOD1^{WT}. Holo-SOD1^{WT} (**A**) or apo- SOD1^{WT} (**B**) (50 μM each) were fully reduced by DTT (5 mM) and then exposed to the indicated concentrations of CSH for 30 min at 37 ºC. After blocking free cysteine groups with iodoacetamide, the protein was separated by a non-reducing 12% SDS-PAGE. Data are representative of three independent experiments.

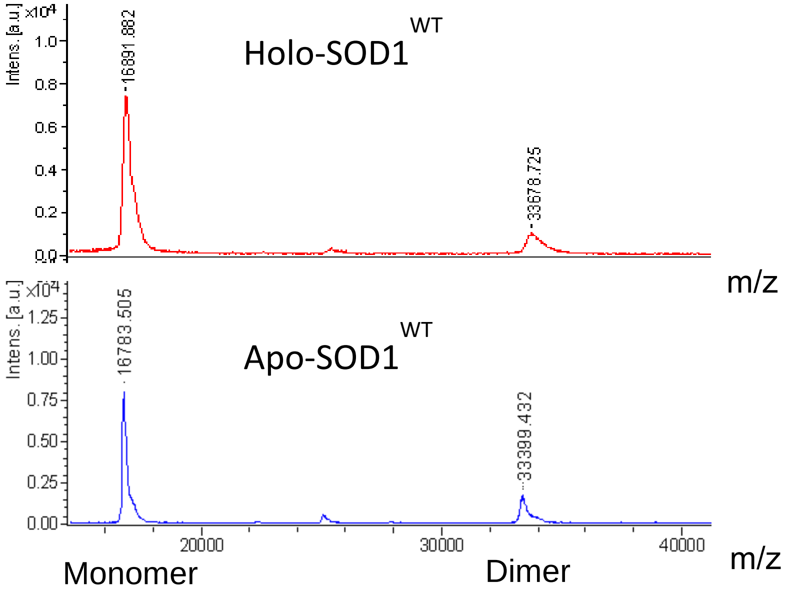

Supplementary Figure S5. The MALDI-TOF mass spectrometry analysis of the metallation state of apo-SOD1^{WT} and reconstituted holo-SOD1^{WT} proteins using the autoflex speed™ MALDI TOF/TOF mass spectrometer and THAP matrix.
